# Polymorphisms in peptidylarginine deiminase associate with rheumatoid arthritis in diverse Asian populations: evidence from MyEIRA study and meta-analysis

**DOI:** 10.1186/ar4093

**Published:** 2012-11-19

**Authors:** Chun Lai Too, Shahnaz Murad, Jasbir Singh Dhaliwal, Per Larsson, Xia Jiang, Bo Ding, Lars Alfredsson, Lars Klareskog, Leonid Padyukov

**Affiliations:** 1 Rheumatology Unit, Department of Medicine, Karolinska University Hospital, CMM L8:04, S-171 76 Stockholm, Sweden; 2 Institute for Medical Research, Jalan Pahang, 50588 Kuala Lumpur, Malaysia; 3 Department of Rheumatology, R 92, Karolinska University Hospital Huddinge, S-171 76 Stockholm, Sweden; 4 Institute of Environmental Medicine, Karolinska Institutet, Nobel väg 13, Box 210, S-171 77 Stockholm, Sweden

## Abstract

**Introduction:**

The majority of our knowledge regarding disease-related mechanisms of uncontrolled citrullination and anti-citrullinated protein antibody development in rheumatoid arthritis (RA) was investigated in Caucasian populations. However, peptidylarginine deiminase (PADI) type 4 gene polymorphisms are associated with RA in East Asian populations and weak or no association was found in Caucasian populations. This study explores the association between the PADI4 polymorphisms and RA risk in a multiethnic population residing in South East Asia with the goal of elucidating generalizability of association in non-Caucasian populations.

**Methods:**

A total of 320 SNPs from the PADI locus (including PADI1, PADI2, PADI3, PADI4 and PADI6 genes) were genotyped in 1,238 RA cases and 1,571 control subjects from the Malaysian Epidemiological Investigation of Rheumatoid Arthritis (MyEIRA) case-control study. Additionally, we conducted meta-analysis of our data together with the previously published studies of RA from East Asian populations.

**Results:**

The overall odds ratio (OR_overall_) for the PADI4 (rs2240340) allelic model was 1.11 (95% confidence interval (CI) = 1.00 to 1.23, *P *= 0.04) and for the genotypic model was 1.20 (95% CI = 1.01 to 1.44, *P *= 0.04). Haplotype analysis for four selected PADI4 SNPs revealed a significant association of one with susceptibility (*P *= 0.001) and of another with a protective effect (*P *= 0.02). The RA susceptibility was further confirmed when combined meta-analysis was performed using these data together with data from five previously published studies from Asia comprising 5,192 RA cases and 4,317 control subjects (OR_overall _= 1.23 (95% CI = 1.16 to 1.31, *P*_heterogeneity _= 0.08) and 1.31 (95% CI = 1.20 to 1.44, *P*_heterogeneity _= 0.32) in allele and genotype-based models, respectively). In addition, we also detected a novel association of PADI2 genetic variant rs1005753 with RA (OR_overall _= 0.87 (95% CI = 0.77 to 0.99)).

**Conclusion:**

Our study demonstrates an association between PADI4 and RA in the multiethnic population from South East Asia and suggests additional association with a PADI2 gene. The study thus provides further support for the notion that polymorphisms in genes for enzymes responsible for citrullination contribute to RA development in multiple populations of Asian descent.

## Introduction

Most studies of the association between rheumatoid arthritis (RA) and genetic factors have focused on a group of human leukocyte antigens in the major histocompatibility complex [1] and a detailed account of the contributions from different major histocompatibility complex genes and their structural correlates was recently published for a number of Caucasian populations [2]. Additional contributions from more than 30 different non-HLA loci have been demonstrated, mainly in populations of Caucasian origin [3]. Important differences for RA susceptibility genes have, however, been described between Caucasian and non-Caucasian populations, as seen both from which HLA alleles are associated with disease [4-7] and from associations with non-HLA genes. A particular interesting difference between Caucasian and Asian populations has been demonstrated in genes from a peptidylarginine deiminase (PADI) locus, where a polymorphism was first demonstrated in a Japanese population [8] and later confirmed in additional Japanese and Korean populations [9-12]. These polymorphisms are of particular interest for the pathogenesis of RA since PADI4 and other PADI enzymes catalyze change from peptidylarginine to peptidylcitrulline, a target of anti-citrullinated protein antibody (ACPA), through a post-translational modification process referred to as citrullination [13,14].

The associations between PADI4 polymorphisms and RA have so far focused on the Japanese and Korean populations [9,11,15]. The effect of PADI4 polymorphisms on RA risk, however, remains unclear in the Han Chinese population [16,17]. An association between PADI4 and RA has also been observed in German and North American populations [18,19], while such an association has not been replicated in other Caucasian populations (for example, British, Spanish, Swedish and Hungarian) [19-23], despite a comparable allele frequency between these Asian and Caucasian populations. The largest study performed in the UK population with over 19,000 subjects found no evidence for association between the PADI4_94 SNP (rs2240340) and RA. In a meta-analysis on previously published European studies together with this UK study, the association between the PADI4_94 genotype and RA was weak and statistically not significant (odds ratio (OR) 1.06, 95% confidence interval (CI) = 0.99 to 1.13, *P *= 0.12) [23]. There is thus a need to further investigate the impact of genetic variations in PADI-associated genes in additional populations. Since ethnic differences are likely to go hand in hand with different environmental exposures, it will be helpful to study it in more divergent populations with some similarities in genetic background.

The PADI gene region is located at chromosome 1p36. This locus contains the cluster of all the PADI genes (PADI1 to PADI4 and PADI6). Of the five isotypes of PAD protein, PAD2 and PAD4 have been reported to be expressed as active enzymes in RA synovium, where citrullination of matrix proteins could potentially create antigenic peptides [24,25] and where increased citrullination has been demonstrated in inflamed synovial tissues [26,27].

In the present study, which was performed in the South East Asia region, the aim was to determine whether the association between the PADI4 polymorphisms and RA risk could be generalized to the Malaysian populations with Malay, Chinese and Indian ethnicity. We also investigated multiple SNPs from the locus (PADI1 to PADI4 and PADI6) in this multiethnic case-control study involving early RA.

## Materials and methods

### Study population

The source of data for our investigation was the multicenter Malaysian Epidemiological Investigation of Rheumatoid Arthritis (MyEIRA) case-control study, comprising 1,238 cases of RA and 1,571 control subjects. The demographic characteristics of RA cases and controls are shown in Table 1. Of the 1,238 cases, 516 (41.7%) were Malays, 255 (20.6%) were Chinese, 379 (30.6%) were Indians and 88 (7.1%) were of other or mixed ethnicities from South East Asia. The details of the MyEIRA study have been described elsewhere [6]. Briefly, all RA cases were diagnosed by rheumatologists according to the 1987 revised American College of Rheumatology criteria [28]. The disease duration for the RA cases was on average 1 year (interquartile range = 2 years). For each potential case, a control subject was randomly selected from the population, taking into consideration the subject's age, sex and residential area. Of the 1,571 controls, 986 (62.8%) were Malays, 206 (13.1%) were Chinese, 285 (18.1%) were Indians and 94 (6.0%) were of other sub-ethnicities. We performed case-control association analyses with regard to the influence from different genetic factors for each ethnic group separately as well as for the meta-analysis where all four ethnic groups were included. The study was approved by the Medical Research and Ethics Committee, Ministry of Health, Malaysia, and written informed consent was obtained from all participants.

**Table 1:** Demographic characteristics of rheumatoid arthritis patients and controls in the MyEIRA study population

Characteristic	Malay		Chinese		Indian		Others	
	
	RA (*n *= 516)	Controls (*n *= 986)	RA (*n *= 255)	Controls (*n *= 206)	RA (*n *= 379)	Controls (*n *= 285)	RA (*n *= 88)	Controls (*n *= 93)
Female	86.8	88.1	83.5	86.9	87.9	84.6	84.1	90.4
Age (years)	46 (11.8)	46 (11.4)	52 (11.2)	52 (11.3)	48 (11.6)	49 (10.6)	49 (12.1)	45 (11.7)
ACPA-positive	60.5	2.4	66.3	3.4	67.3	2.1	70.5	1.1
IgM RF-positive	47.5	3.8	49.4	3.4	55.9	5.3	62.5	6.4
IgG RF-positive	48.4	6.3	49.4	6.8	54.1	6.3	64.8	7.4
DRB1 SE-positive^a^	35.5	13.1	36.5	12.1	48.5	30.2	40.9	17.0

Data presented as percentage or mean (standard deviation). ACPA, anti-citrullinated protein antibody; MyEIRA, Malaysian Epidemiological Investigation of Rheumatoid Arthritis; RA, rheumatoid arthritis; RF, rheumatoid factor; SE, shared epitope. ^a^Patients and controls carrying one or two alleles of a SE were classified as SE-positive.

### DNA extraction, selection of markers and genotyping

The genomic DNA was extracted using the QIAamp DNA Blood Mini Kit (Qiagen, Hilden, Germany). All DNA samples were stored at -20°C until testing. We investigated 320 SNPs selected from the PADI locus on Immunochip and from other studies [8,19-21,29]. The list of the genotyped PADI SNPs is presented in Table S1 in Additional File 1. The SNPs were genotyped either using the TaqMan SNP genotyping assay (Applied Biosystems, Foster City, CA, USA) or by the Illumina iSelect HD custom genotyping array (Immunochip, Illumina, Inc, San Diego, CA, USA). SNPs with call rates <95%, monomorphic SNPs, SNPs with minor allele frequency <0.005, and SNPs with Hardy-Weinberg equilibrium *P *<1.0 × 10^-4 ^in RA cases and controls were excluded from statistical evaluation. This resulted in 227, 238, 263 and 248 SNPs passing quality-control filters for Malay, Chinese, Indian and other or mixed ethnicities, respectively. Genetic outliers for each ethnic group (that is, Malay, four cases and two controls; Chinese, five cases and four controls; Indian, 10 cases) were removed after principal component analysis to correct for possible population stratification.

To assess genotyping robustness, comparisons were made between the TaqMan assay and the Illumina iSELECT HD custom genotyping array (Immunochip) assay regarding PADI4_94 (rs2240340), which resulted in a 100% match of the genotyping calls for both RA cases and controls.

Genotyping for HLA-DRB1 shared epitope (SE) alleles - defined by DRB1*01:01, DRB1*01:02, DRB1*01:07, DRB1*04:01, DRB1*04:04, DRB1*04:05, DRB1*04:08, DRB1*04:10, DRB1*10:01 and DRB1*10:03 alleles - was performed by HLA-DRB1 high-resolution sequence-specific oligonucleotide PCR for all the cases and controls as described previously [6]. Individuals carrying one or two SE alleles were classified as SE-positive [6].

### Autoantibody measurements

ACPA and rheumatoid factors were identified and measured with ELISA kits (Immunoscan RA Mark 2; Euro-Diagnostica, Malmö, Sweden) as described elsewhere [6]. Antibody levels ≥25 AU/ml and >15 IU/ml were regarded as ACPA-positive and rheumatoid factor-positive, respectively.

### Statistical analysis

Genotype, allele and haplotype frequencies were assessed with Yate's chi-square and/or Fisher's exact test when appropriate by means of IBM SPSS Statistics 20.0 software (SPSS Inc., Chicago, USA). The frequencies of the alleles and genotypes of PADI SNPs were compared between RA cases and control subjects and ORs with 95% CIs were calculated. Haplotype analysis was carried out by Haploview [30]. Power calculation was performed for a one-tail test at a significance level of 0.05. For meta-analysis, the Mantel-Haenszel method was employed with a fixed-effects model and 95% CI for cumulative or overall odds ratio [31]. The significance of the cumulative OR was determined by the Z-test. The between-study heterogeneity was assessed using the Cochran Q-statistic (*P *<0.10 considered significant). In addition, the *I*^2 ^metric [*I*^2 ^= (Q - df)/Q] was used to describe the percentage of variation across the studies due to heterogeneity. *I*^2 ^values of 25%, 50% and 75% were assigned as low, moderate and high estimates, respectively.

## Results

### Characteristics of RA cases and controls

Among the RA cases, the overall mean ± standard deviation age was 48 ± 11.6 years, 86.3% were female, 64.5% were ACPA-positive, 40.1% were HLA-DRB1 SE-positive, and 51.5% were rheumatoid factor-positive. The mean ± standard deviation age of control subjects was 47 ± 11.4 years and 87.5% were female. The distribution of ethnic groups (Malay, Chinese, Indian and other or mixed ethnicities) is shown in Table 1.

### Evaluation of the PADI gene polymorphisms and RA association in the MyEIRA study

Collectively, our study has 88% statistical power to detect genotype/minor allele frequency differences of the magnitude reported in the initial positive study in a Japanese population [8].

Overall, the single-point analyses of variations in the PADI1, PADI2, PADI3, PADI4 and PADI6 genes revealed a modest genetic effect size in the RA population and showed that the peak association varies between different ethnic groups. For example, the rs2526839 variant showed the peak association signal in Malay RA patients (OR = 1.24, 95% CI = 1.04 to 1.43, *P *= 0.0126), the rs3003444 variant in Chinese RA patients (OR = 1.60, 95% CI = 1.20 to 2.13, *P *= 0.0013) and the rs113475583 variant in Indian RA patients (OR = 1.91, 95% CI = 1.19 to 3.07, *P *= 0.0064) (see Figure S1 in Additional File 1).

### PADI2 gene polymorphism as a risk factor in RA development

**Figure 1:**
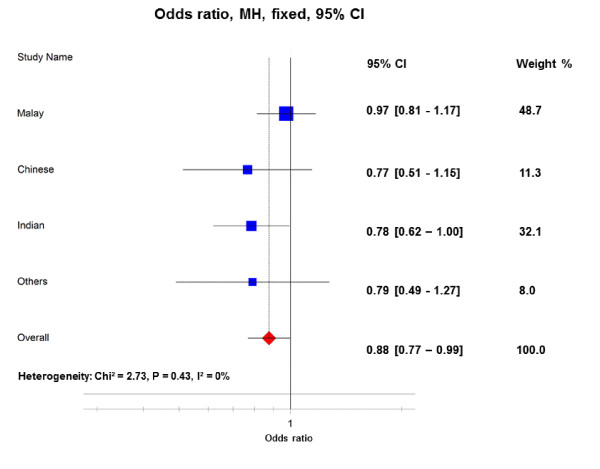
**Allelic association between PADI2 rs1005753 polymorphism and risk of rheumatoid arthritis in the MyEIRA study**. Meta-analysis of the minor allele (rs1005753_G) versus the common allele (rs1005753_T). CI, confidence interval; MH, Mantel-Haenszel; p_het_, *P *value for heterogeneity; *I*^2^, degree of heterogeneity (%); MyEIRA, Malaysian Epidemiological Investigation of Rheumatoid Arthritis; PADI, peptidylarginine deiminase.

The PADI2 gene encodes PAD2 enzyme, which is the most widely expressed family member of PAD. An association between RA and the PADI2 variant was found in a Korean population [32]. We undertook this study for further investigation of whether PADI2 polymorphisms are also at risk for RA development in three independent Asian populations. Interestingly, the PADI2 variant meta-analysis demonstrated a possible novel association of the PADI2 rs1005753 variant with RA, both in the allelic model (OR_overall _= 0.88, 95% CI = 0.77 to 0.99, *P *= 0.04) and in the dominant/recessive genotype model (OR_overall _= 0.84, 95% CI = 0.72 to 0.99, *P *= 0.04) (Figure 1). In this multiethnic case-control study, no evidence of heterogeneity was found for the PADI2 rs1005753 variant (*P*_heterogeneity _= 0.43, *I*^2 ^= 0%).

### Generalizability of PADI4 gene polymorphisms as a risk factor in RA development

**Figure 2:**
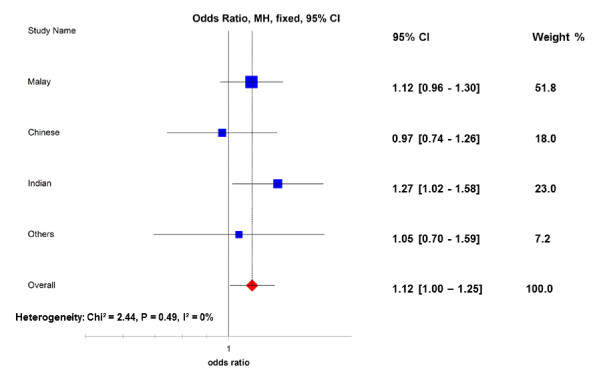
**Allelic association between PADI4 rs2240340 polymorphism and risk of rheumatoid arthritis in the MyEIRA study**. Meta-analysis of the minor allele (rs2240340_T) versus the common allele (rs2240340_C). CI, confidence interval; MH, Mantel-Haenszel; p_het_, *P *value for heterogeneity; *I*^2^, degree of heterogeneity (%); MyEIRA = Malaysian Epidemiological Investigation of Rheumatoid Arthritis; PADI, peptidylarginine deiminase.

A summary of the MyEIRA meta-analysis for the PADI4 polymorphism with RA is presented in Figure 2. The MyEIRA meta-analysis suggests an association of the PADI4 rs2240340 variant with RA also found in the Malaysian population (OR_overall _= 1.11, 95% CI = 1.00 to 1.23, *P *= 0.04). The meta-analysis also revealed a significantly increased OR (OR_overall _= 1.20, 95% CI = 1.01 to 1.44, *P *= 0.04) when applying a dominant/recessive genotype model for the PADI4 rs2240340 variant, suggesting that the PADI4 is associated with RA in the Malaysian population of Asian descent.

To clarify the role of the PADI4 gene as a possible susceptibility factor for RA development in different Asian ethnic populations, we performed a combined meta-analysis using our current data together with the previously published studies from Asia. We searched information from the PubMed database, ISI Web of Knowledge and Google. Six published original case-control studies related to PADI4 polymorphisms in RA in three different Asian populations were identified: three Japanese studies including the first positive report, one Korean study and two Chinese studies [8-11,16,17]. All studies had used the defined American College of Rheumatology criteria [28] for the diagnosis of RA as compared with our study. The strongest evidence of association reported in the first study was given by PADI4_94 (rs2240340); we therefore restricted the current combined meta-analysis to this genetic variant. Of the six published papers, the Korean study did not genotype for PADI4 rs2240340. Instead, we used genotype data of PADI4_89 (rs11203366). This choice was made because these SNPs are in strong linkage disequilibrium (*r*^2 ^= 1.00) and the minor allele frequency of these SNPs are nearly equal according to the International Human Genome Project Chinese and Japanese populations (International HapMap CHB + JPT) (Asian population). Five studies were included in the combined meta-analysis together with our new data for PADI4 rs2240340 [8-11,16]. The PADI4 SNPs investigated in Fan and colleagues' study were not analyzed because they exhibited significant deviation from Hardy-Weinberg equilibrium (*P *<0.001) [17], which questions the validity of the genotyping data.

**Figure 3:**
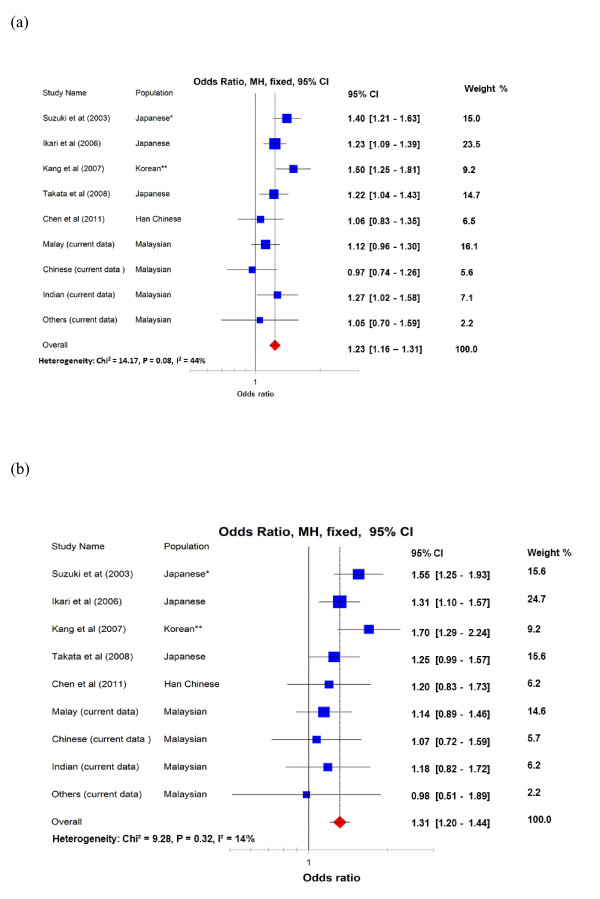
**Allelic and genotypic association between PADI4 rs2240340 polymorphism and rheumatoid arthritis in Asian populations**. Combined meta-analysis of **(a) **the allelic model and **(b) **the genotypic model between PADI4 rs2240340 polymorphism and risk of developing RA in Asian populations. (a) *If the first Japanese study was excluded, overall odds ratio (OR_overall_) = 1.21 (1.13 to 1.29), p_het _= 0.15, *I*^2 ^= 35%; **if the Korean study was excluded as the PADI4 rs2240340 SNP was not investigated, OR_overall _= 1.21 (1.13 to 1.29), p_het _= 0.23, *I*^2 ^= 25%; if both the first Japanese study and the Korean study were excluded, OR_overall _= 1.17 (1.09 to 1.25), p_het _= 0.58, *I*^2 ^= 0%. (b) *If the first Japanese study was excluded, OR_overall _= 1.27 (1.15 to 1.40), p_het _= 0.47, *I*^2 ^= 0%; **if the Korean study was excluded as the PADI4 rs2240340 SNP was not investigated, OR_overall _= 1.28 (1.16 to 1.40), p_het _= 0.58, *I*^2 ^= 0%; if both the first Japanese study and the Korean study were excluded, OR_overall _= 1.22 (1.10 to 1.35), p_het _= 0.93, *I*^2 ^= 0%. Minor allele (rs2240340_T) versus common allele (rs2240340_C). CI, confidence interval; MH, Mantel-Haenszel; p_het_, *P *value for heterogeneity; *I*^2^, degree of heterogeneity expressed as percentage; RA, rheumatoid arthritis.

In total, the combined meta-analysis of the PADI4 rs2240340 included 5,192 RA cases and 4,317 control subjects from Asian populations. The Mantel-Haenszel method with fixed effects exhibited significant ORs in both the allelic model (OR = 1.23, 95% CI = 1.16 to 1.31, *P *<10^-4^) and the dominant/recessive genotype model (OR = 1.31, 95% CI = 1.20 to 1.44, *P *<10^-4^) (Figure 3a, b). A moderate level of between-study heterogeneity was observed in the allele model (*P*_heterogeneity _= 0.08, *I*^2 ^= 44%), but not in the genotype model (*P*_heterogeneity _= 0.32, *I*^2 ^= 14%). With the exclusion of the initial positive report, which tends to suggest a stronger genetic effect (winner's curse) than those in subsequent studies [9-11,16], the cumulative OR decreased slightly but remained highly significant (*P *<10^-4^) with no significant heterogeneity (*P*_heterogeneity _= 0.47, *I*^2 ^= 0%) (see Figure 3 caption).

### Stratified analysis

Stratified analyses were performed to detect association between the PADI gene variants and RA risk within specific population subgroups as well as in relation to more homogeneous subsets of RA cases. Stratifying by sex, carriage of SE alleles, presence of ACPA or presence of rheumatoid factors among the cases revealed no evidence of different associations between the compared subgroups (data not shown). Nevertheless, it is noteworthy that a set of different SNPs variants were associated with risk of developing ACPA-positive and ACPA-negative RA within specific subpopulations, although the results were not significant after correction for multiple comparisons (Table 2). It would be interesting to investigate the association between RA and these SNP variants in other populations with more data.

**Table 2:** Significant SNP variants associated with ACPA-positive and ACPA-negative RA in the MyEIRA study population

Ethic group	SNP variant	Risk allele	Ratio counts (case, control)	Frequencies (case, control)	**χ**^**2**^	*P *value
ACPA-positive RA						
Malay	rs12042956	G	50:562, 103:1819	0.082, 0.054	6.465	0.011
Malay	rs7538876	G	496:122, 1480:478	0.803, 0.756	5.738	0.0166
Malay	rs2526839	C	240:378, 667:1291	0.388, 0.341	4.684	0.0304
Chinese	rs3003444	A	247:83, 262:142	0.748, 0.649	8.539	0.0035
Chinese	rs2977299	G	223:107, 235:169	0.676, 0.582	6.851	0.0089
Chinese	rs3766298	A	223:107, 234:168	0.676, 0.582	6.779	0.0092
Indian	rs113475583	G	481:21, 532:44	0.958, 0.924	5.653	0.0174
Indian	rs72633848	A	483:21, 536:44	0.958, 0.924	5.594	0.018
Indian	rs35903413	G	498:6, 564:16	0.988, 0.972	3.335	0.0678
ACPA-negative RA						
Malay	rs16825533	G	67:337, 227:1731	0.166, 0.116	7.654	0.0057
Malay	rs57744451	G	67:339, 234:1724	0.165, 0.120	6.269	0.0123
Malay	rs2240337	G	388:14, 1826:128	0.965, 0.934	5.541	0.0186
Chinese	rs3003444	A	125:43, 262:142	0.744, 0.649	4.949	0.0261
Chinese	rs12037558	A	113:53, 237:167	0.681, 0.587	4.395	0.036
Chinese	rs2977296	G	145:23, 320:84	0.863, 0.792	3.935	0.0473
Indian	rs12042956	A	223:13, 506:64	0.945, 0.888	6.319	0.0119
Indian	rs2977305	A	242:4, 550:26	0.984, 0.955	4.088	0.0432
Indian	rs34324150	G	242:4, 552:26	0.984, 0.955	4.058	0.044

Top three significant SNP variants associated with risk of developing anti-citrullinated protein autoantibody (ACPA)-positive and ACPA-negative rheumatoid arthritis (RA) in three major ethnic groups from the Malaysian Epidemiological Investigation of Rheumatoid Arthritis (MyEIRA) study population

### Haplotype analysis and association test

We first performed the univariate single-population analyses for the association of PADI SNPs with RA, and subsequently those SNPs with statistical significant effects were selected for further haplotype analysis. However, we excluded the SNPs with inconsistent effects within the four ethnic groups, and also those SNPs with a significant level not low enough (*P *<0.05) in one or more ethnic groups. This analysis strategy led us to four PADI4 SNPs for haplotype analysis (rs79907974, rs2240340, rs1748021 and rs2240337). When haplotypes were constructed using these four PADI4 SNPs, seven haplotypes with frequency >1% out of 16 expected haplotypes were found in the study populations (Table 3). Haplotype GCGG was omitted from the association test because it was only found in the Indian ethnic group with a frequency of 5%. When meta-analyses for different ethnic groups in our study were conducted, ATAA and ATAG showed a significant difference between RA cases and controls (*P *= 0.02 and *P *= 0.001, respectively; Table 3) with no significant heterogeneity (*P*_heterogeneity _= 0.78, *I*^2 ^= 0%). These haplotypes represent either protective (ATAA) or susceptible (ATAG) variants. We further tested these haplotype associations with different RA subsets defined by ACPA status. The results showed consistent effects in the RA subsets, and the OR_overall _was comparable between ACPA-positive and ACPA-negative RA subsets (see Table S2 in Additional File 1).

**Table 3:** Haplotype frequencies and meta-analysis of PADI4 polymorphisms in relation to RA in the MyEIRA study

Block	Ethnicity	Haplotype frequency	Ratio counts (case, control)	Frequencies (case, control)	**χ**^**2**^	*P *value	OR (95% CI)	Meta-analysis	***P***_**het**_	*I*^2 ^(%)
ACGG	Malay	0.501	510.8:537.2, 984.2:951.8	0.487, 0.508	1.195	0.2744	0.92 (0.79 to 1.07)	0.91 (0.82 to 1.02), *Z *= 1.62 (*P *= 0.10)	0.64	0
	Chinese	0.567	288.8:217.2, 224.7:175.3	0.571, 0.562	0.076	0.7835	1.04 (0.80 to 1.35)			
	Indian	0.413	292.7:453.3, 246.1:313.9	0.392, 0.439	2.919	0.0875	0.83 (0.66 to 1.03)			
	Others	0.46	78.8:97.2, 88.6:99.4	0.447, 0.471	0.204	0.6519	0.91 (0.60 to 1.37)			

ATAA	Malay	0.055	42.5:1,005.5, 120.7:1,815.3	0.041, 0.062	6.226	0.0126	**0.64 (0.45 to 0.92)**	**0.71 (0.54-0.94)**, *Z *= 2.42 (*P *= 0.02)	0.78	0
	Chinese	0.064	29.0:477.0, 29.0:371.0	0.057, 0.073	0.86	0.3537	0.78 (0.46 to 1.32)			
	Indian	0.015	9.7:736.3, 9.3:550.7	0.013, 0.017	0.293	0.5886	0.83 (0.34 to 2.06)			
	Others	0.039	7.3:168.7, 7.0:181.0	0.042, 0.037	0.046	0.8296	1.07 (0.37 to 3.12)			

ATAG	Malay	0.287	333.6:714.4, 522.9:1,413.1	0.318, 0.270	7.736	0.0054	**1.26 (1.07 to 1.49)**	**1.21 (1.08-1.36)**, *Z *= 3.20 (*P *= 0.001)	0.78	0
	Chinese	0.274	140.8:365.2, 107.1:292.9	0.278, 0.268	0.128	0.7202	1.06 (0.79 to 1.42)			
	Indian	0.398	312.4:433.6, 207.6:352.4	0.419, 0.371	3.09	0.0788	1.22 (0.97 to 1.52)			
	Others	0.34	62.5:113.5, 61.4:126.6	0.355, 0.326	0.336	0.562	1.16 (0.75 to 1.79)			

ATGG	Malay	0.096	108.7:939.3, 178.8:1,757.2	0.104, 0.092	1.001	0.317	1.14 (0.89 to 1.46)	1.02 (0.77-1.36), *Z *= 0.14 (*P *= 0.89)	0.24	29
	Chinese	0.046	23.2:482.8, 18.3:381.7	0.046, 0.046	0.0	0.9908	1.01 (0.54 to 1.90)			
	Indian	0.049	39.1:706.9, 25.3:534.7	0.052, 0.045	0.351	0.5538	1.18 (0.71 to 1.97)			
	Others	0.091	11.0:165.0, 22.0:166.0	0.063, 0.117	3.268	0.0706	0.50 (0.24 to 1.07)			

GTAG	Malay	0.041	38.6:1,009.4, 83.8:1,852.2	0.037, 0.043	0.714	0.398	0.85 (0.58 to 1.26)	0.94 (0.72-1.21), *Z *= 0.49 (*P *= 0.63)	0.59	0
	Chinese	0.042	20.1:485.9, 17.9:382.1	0.040, 0.045	0.138	0.7099	0.87 (0.46 to 1.67)			
	Indian	0.065	47.9:698.1, 37.1:522.9	0.064, 0.066	0.023	0.8798	0.97 (0.62 to 1.51)			
	Others	0.046	10.4:165.6, 6.4:181.6	0.059, 0.034	1.326	0.2496	1.83 (0.65 to 5.14)			

CI, confidence interval; *I*^2^, degree of heterogeneity (%); MyEIRA, Malaysian Epidemiological Investigation of Rheumatoid Arthritis; OR, odds ratio; PADI, peptidylarginine deiminase; *P*_het_, *P *value for heterogeneity; RA, rheumatoid arthritis.

## Discussion

In this study, we used a multiethnic population of Asian descent in Malaysia to determine the association between PADI4 polymorphisms and risk of RA, and convincingly validated this association. Our current data, together with the previously published data on Asian populations, strongly support PADI4 as a RA susceptibility gene in different ethnic populations of Asian descent. Collectively, our data extend previous results on PADI4 and RA based on Asian populations, mainly in Japanese and Korean populations [8-11,15,33], to be observed in another population of Asian origin (that is, Malay, Chinese and Indian ethnicity). Notably, a possible novel association between the PADI2 genetic variant and RA risk was also found in the multiethnic Malaysian population.

The combined meta-analysis was performed regardless of the ACPA status, since such data were not available in the previous published papers. However, our present study showed a comparable minor allele frequency between the RA subsets defined by ACPA status, thus suggesting that the risk from PADI locus is likely to be common for the two main subgroups of RA.

Previously, the association studies of the PADI4 polymorphism and RA found in Asian populations were predominantly focused on Japanese and Korean populations [8-11,15], which are geographically and historically closely related and genetically quite similar to each other [34]. The results, however, were inconsistent across two independent studies in Han Chinese populations [16,17]. Malaysia is a multiethnic country in South East Asia representing genetic diversity across multiple large ethnic populations of Asian origin (that is, Malays, Chinese and Indians). In our study, we were able to address the question of whether PADI4 polymorphisms confer a risk of RA in this diverse population. We found that the PADI4_94 polymorphism is associated with an increased risk of developing RA in this population. Interestingly, as can be seen from Figure 3, the effect for the Han Chinese population is probably lower than for other Asian populations, which may be an explanation for previous inconsistency in published data.

Haplotype analysis did not reveal a higher effect size in comparison with univariate SNP analyses in our study. Nevertheless, meta-analysis of PADI4 haplotypes with the current data implied that association with RA was probably driven by the rs2240337 variant. This genetic variant, however, has low minor allele frequency in our materials (that is, minor allele frequency in Malay = 0.059, Chinese = 0.066, Indian = 0.032 and others = 0.042). Noteworthy is that this SNP variant, on the contrary, is in complete linkage disequilibrium (*D*' = 1.00) with the PADI4 SNP rs766499 variant, which was reported recently to be associated with the Japanese RA population at a genome-wide level of significance in meta-analysis [12].

An additional novel finding in our study is the discovery of a possible association between the PADI2 genetic variant (rs1005753) and RA, which was observed both in allele and genotype models. The genetic effect observed is possibly due to an indirect association, where the identified genetic variants (that is, PADI2) by themselves are not functional but are in linkage disequilibrium with a causal variant polymorphism such as the PADI4 gene. However, this would be unlikely as the PADI2 genetic variant (rs1005753) did not show any linkage disequilibrium with the numerous investigated SNPs spanning between PADI1 and PADI6 in the present study. We observed *r*^2 ^<0.01 for all ethnic groups studied. A previous study by Freudenberg and colleagues reported an association between the PADI2 genetic variant (rs2075696) and RA in a Korean study [32]. Interestingly, the rs1005753 variant associated in our study and the rs2075696 variant are located in two different linkage disequilibrium blocks in the PADI2 gene locus. The International HapMap CHB+JPT project showed no linkage disequilibrium relationship between these two SNPs (*D*' = 0.0050, *r*^2 ^= 0.0). Together, our data may suggest a possible risk effect between the rs1005753 variant and RA in the Malaysian population.

Worthy of mention is that PADI2 and PADI4 are the only two genes that are highly expressed in hemapoetic cells [35]. In RA, the expression levels of PAD2 and PAD4 were correlated with the intensity of inflammation, and both enzymes were demonstrable within or in the vicinity of citrullinated fibrins deposits [36]. The PADI2 gene encodes the PAD2 enzyme, is perhaps playing a role in RA pathogenesis on its own, independently from PADI4. Nevertheless, it is important to perform further extension of the study to gain better statistical power to investigate this association in a single-population analysis and further to replicate the association in independent cohorts of Asian origin. Since the difference between Caucasian and Asian populations may represent both genetic and environmental heterogeneity, it is logical to propose a gene-environmental interaction study for PADI genes in the development of RA.

## Conclusion

This study demonstrates an association between PADI4 polymorphisms and RA in the Malaysian population. The currently updated combined meta-analysis of Asian populations further supports the hypothesis that the PADI locus contributes to the development of RA in different Asian populations and that this genetic effect is generalized to multiple ethnic populations of Asian descent.

## Abbreviations

ACPA: anti-citrullinated protein antibody; CI: confidence interval; ELISA: enzyme-linked immunosorbent assay; HLA: human leukocyte antigen; *I*^2^: degree of heterogeneity; International HapMap CHB+JPT: International Human Genome Project Chinese and Japanese; MyEIRA: Malaysian Epidemiological Investigation of Rheumatoid Arthritis; OR: odds ratio; PADI: peptidylarginine deiminase; PCR: polymerase chain reaction; RA: rheumatoid arthritis; SE: shared epitope; SNP: single nucleotide polymorphism.

## Competing interests

The authors declare that they have no competing interests.

## Authors' contributions

TCL and LP had full access to all data in this study and take responsibility for the integrity of the data and the accuracy of the data analysis. TCL and LP drafted the manuscript, performed statistical analysis and manuscript preparation. TCL, SM, JSD, PL, XJ, BD, LA, LK and LP conceived the study and participated in the design of the study and manuscript editing. TCL and LP performed serological and molecular genetics assay. TCL and LP take responsibility for the acquisition of data, analysis and interpretation of data. All authors were involved in revising the paper critically for important intellectual content, and all authors approved the final version to be published.

## Supplementary Material

Additional file 1 **Table S1 presenting a list of PADI SNPs investigated in the MyEIRA study population**. A complete set of 320 SNPs selected from the PADI locus on Immunochip and from other studies. The SNPs were genotyped either using the TaqMan SNP genotyping assay (Applied Biosystems, USA) or by Illumina iSELECT HD custom genotyping array (Immunochip). Table S2 presenting the haplotype frequencies and meta-analysis of PADI4 polymorphisms in the MyEIRA study by ACPA status. The haplotype analysis and meta-analysis of PADI4 polymorphisms in the MyEIRA study were performed in different subsets of RA defined by ACPA status. Bold results indicate significant association between the PADI haplotypes and subsets of RA. Figure S1 showing the regional association plots with recombination rate on the PADI genes for the three major ethnic groups from the MyEIRA study. Regional association plots on the PADI genes including PADI1, PADI2, PADI3, PADI4 and PADI6 for the three major ethnic groups from MyEIRA study showing the peak association in each ethnic group. Graphs centered on the most significant SNP in each ethnic group. The *r*^2 ^values (linkage disequilibrium between the most significant SNP and the rest of SNPs in the region) are calculated on the MyEIRA data and the recombination rates are based on the International HapMap CHB+JPT data. Click here for file
