# Supplementary material for: Polymorphisms in peptidylarginine deiminase associate with rheumatoid arthritis in diverse Asian populations: evidence from MyEIRA study and meta-analysis
Source: Arthritis Res Ther. 2012 Nov 19;14(6):R250. doi: 10.1186/ar4093 (PMC3674620; doi:10.1186/ar4093)

**Additional File 1**

**Table S1.** List of PADI SNPs investigated in MyEIRA study population

| ***SNP Name*** | ***SNP Position (GRChr37)*** | ***GeneSymbol*** | ***SNP filtering Quality control passed (Y)/failed (N)*** | | | ***Genotyping method*** |
| --- | --- | --- | --- | --- | --- | --- |
|  |  |  | ***Malay*** | ***Chinese*** | ***Indian*** |  |
| *rs2235927* | *17394775* | *PADI2* | *Y* | *Y* | *Y* | *TaqMan assay* |
| *rs2647199* | *17403563* | *PADI2* | *Y* | *Y* | *Y* | *TaqMan assay* |
| *rs2235917* | *17422998* | *PADI2* | *Y* | *Y* | *Y* | *TaqMan assay* |
| *rs1005753* | *17444769* | *PADI2* | *Y* | *Y* | *Y* | *TaqMan assay* |
| *rs12026071* | *17560646* | *PADI1* | *Y* | *Y* | *Y* | *Immunochip* |
| *rs2293916* | *17597655* | *PADI3* | *Y* | *Y* | *Y* | *Immunochip* |
| *rs4595409* | *17597721* | *PADI3* | *Y* | *Y* | *Y* | *Immunochip* |
| *rs76899920* | *17598168* | *PADI3* | *N* | *N* | *N* | *Immunochip* |
| *rs75197044* | *17598575* | *PADI3* | *Y* | *Y* | *Y* | *Immunochip* |
| *rs16824705* | *17598684* | *PADI3* | *Y* | *Y* | *Y* | *Immunochip* |
| *rs2977293* | *17599444* | *PADI3* | *Y* | *Y* | *Y* | *Immunochip* |
| *rs74705609* | *17599467* | *PADI3* | *Y* | *Y* | *Y* | *Immunochip* |
| *rs3003433* | *17599525* | *PADI3* | *N* | *N* | *Y* | *Immunochip* |
| *rs12079310* | *17599539* | *PADI3* | *N* | *N* | *Y* | *Immunochip* |
| *rs11584781* | *17599897* | *PADI3* | *N* | *N* | *Y* | *Immunochip* |
| *rs12037653* | *17600317* | *PADI3* | *Y* | *Y* | *Y* | *Immunochip* |
| *rs2977295* | *17600437* | *PADI3* | *Y* | *Y* | *Y* | *Immunochip* |
| *rs2977296* | *17600469* | *PADI3* | *Y* | *Y* | *Y* | *Immunochip* |
| *rs11203346* | *17600822* | *PADI3* | *Y* | *Y* | *Y* | *Immunochip* |
| *rs2293917* | *17601083* | *PADI3* | *Y* | *Y* | *Y* | *Immunochip* |
| *rs11585357* | *17601165* | *PADI3* | *Y* | *Y* | *Y* | *Immunochip* |
| *rs2293918* | *17601269* | *PADI3* | *N* | *N* | *N* | *Immunochip* |
| *rs2293919* | *17601355* | *PADI3* | *N* | *N* | *N* | *Immunochip* |
| *rs3766298* | *17602087* | *PADI3* | *Y* | *Y* | *Y* | *Immunochip* |
| *rs3003434* | *17602191* | *PADI3* | *Y* | *Y* | *Y* | *Immunochip* |
| *rs12135460* | *17602225* | *PADI3* | *N* | *N* | *N* | *Immunochip* |
| *rs2977298* | *17602366* | *PADI3* | *N* | *N* | *N* | *Immunochip* |
| *rs3003435* | *17602386* | *PADI3* | *Y* | *Y* | *Y* | *Immunochip* |
| *rs79270532* | *17602542* | *PADI3* | *Y* | *Y* | *Y* | *Immunochip* |
| *rs80012763* | *17603436* | *PADI3* | *Y* | *Y* | *Y* | *Immunochip* |
| *rs72646785* | *17603472* | *PADI3* | *Y* | *Y* | *Y* | *Immunochip* |
| *rs11588043* | *17603699* | *PADI3* | *Y* | *Y* | *Y* | *Immunochip* |
| *rs72646786* | *17604437* | *PADI3* | *Y* | *Y* | *Y* | *Immunochip* |
| *rs2977299* | *17604701* | *PADI3* | *Y* | *Y* | *Y* | *Immunochip* |
| *rs3753375* | *17605547* | *PADI3* | *Y* | *Y* | *Y* | *Immunochip* |
| *rs11585118* | *17605774* | *PADI3* | *Y* | *Y* | *Y* | *Immunochip* |
| *rs11585119* | *17605792* | *PADI3* | *Y* | *Y* | *Y* | *Immunochip* |
| *rs114580042* | *17606189* | *PADI3* | *N* | *N* | *N* | *Immunochip* |
| *rs3003438* | *17606560* | *PADI3* | *Y* | *Y* | *Y* | *Immunochip* |
| *rs3003439* | *17606572* | *PADI3* | *N* | *N* | *N* | *Immunochip* |
| *rs2272630* | *17607068* | *PADI3* | *Y* | *Y* | *Y* | *Immunochip* |
| *rs34097903* | *17607274* | *PADI3* | *N* | *N* | *Y* | *Immunochip* |
| *rs11584287* | *17607501* | *PADI3* | *Y* | *Y* | *Y* | *Immunochip* |
| *rs34826223* | *17607656* | *PADI3* | *N* | *N* | *N* | *Immunochip* |
| *rs115220982* | *17607945* | *PADI3* | *N* | *N* | *N* | *Immunochip* |
| *rs12037558* | *17608353* | *PADI3* | *Y* | *Y* | *Y* | *Immunochip* |
| *rs3766301* | *17608545* | *PADI3* | *Y* | *Y* | *Y* | *Immunochip* |
| *rs3003440* | *17608680* | *PADI3* | *Y* | *Y* | *Y* | *Immunochip* |
| *rs115548670* | *17608954* | *PADI3* | *N* | *N* | *Y* | *Immunochip* |
| *rs35624745* | *17609432* | *PADI3* | *N* | *N* | *N* | *Immunochip* |
| *rs2293920* | *17609448* | *PADI3* | *Y* | *Y* | *Y* | *Immunochip* |
| *rs16824888* | *17609745* | *PADI3* | *Y* | *Y* | *Y* | *Immunochip* |
| *rs72646789* | *17610030* | *PADI3* | *Y* | *Y* | *Y* | *Immunochip* |
| *rs59877223* | *17610156* | *PADI3* | *Y* | *Y* | *Y* | *Immunochip* |
| *rs12135399* | *17610300* | *PADI3* | *Y* | *Y* | *Y* | *Immunochip* |
| *rs17424382* | *17610388* | *PADI3* | *N* | *N* | *N* | *Immunochip* |
| *rs12743888* | *17610395* | *PADI3* | *N* | *N* | *N* | *Immunochip* |
| *rs2977300* | *17610466* | *PADI3* | *Y* | *Y* | *Y* | *Immunochip* |
| *rs12744056* | *17610532* | *PADI3* | *N* | *N* | *N* | *Immunochip* |
| *rs2977301* | *17610602* | *PADI3* | *Y* | *Y* | *Y* | *Immunochip* |
| *rs3750304* | *17610899* | *PADI3 | PADI4* | *Y* | *Y* | *Y* | *Immunochip* |
| *rs2977302* | *17610998* | *PADI3 | PADI4* | *Y* | *Y* | *Y* | *Immunochip* |
| *rs72646792* | *17611209* | *PADI3 | PADI4* | *Y* | *Y* | *Y* | *Immunochip* |
| *rs12043098* | *17611257* | *PADI3 | PADI4* | *Y* | *Y* | *N* | *Immunochip* |
| *rs10492990* | *17611288* | *PADI3 | PADI4* | *Y* | *Y* | *Y* | *Immunochip* |
| *rs6684387* | *17611519* | *PADI3 | PADI4* | *Y* | *Y* | *Y* | *Immunochip* |
| *rs2977303* | *17611548* | *PADI3 | PADI4* | *Y* | *Y* | *Y* | *Immunochip* |
| *rs2977304* | *17611615* | *PADI3 | PADI4* | *Y* | *Y* | *Y* | *Immunochip* |
| *rs11808782* | *17612706* | *PADI3 | PADI4* | *N* | *N* | *Y* | *Immunochip* |
| *rs3003444* | *17613066* | *PADI3 | PADI4* | *Y* | *Y* | *Y* | *Immunochip* |
| *rs113593167* | *17613355* | *PADI3 | PADI4* | *Y* | *Y* | *Y* | *Immunochip* |
| *rs2977305* | *17613737* | *PADI3 | PADI4* | *Y* | *Y* | *Y* | *Immunochip* |
| *rs72646797* | *17613838* | *PADI3 | PADI4* | *Y* | *Y* | *Y* | *Immunochip* |
| *rs3003445* | *17613905* | *PADI3 | PADI4* | *N* | *N* | *N* | *Immunochip* |
| *rs72646798* | *17614422* | *PADI3 | PADI4* | *Y* | *Y* | *Y* | *Immunochip* |
| *rs114311271* | *17614492* | *PADI3 | PADI4* | *Y* | *Y* | *Y* | *Immunochip* |
| *rs3123529* | *17615359* | *PADI3 | PADI4* | *Y* | *Y* | *Y* | *Immunochip* |
| *rs115353201* | *17615382* | *PADI3 | PADI4* | *N* | *N* | *Y* | *Immunochip* |
| *rs114195833* | *17615542* | *PADI3 | PADI4* | *Y* | *Y* | *Y* | *Immunochip* |
| *rs3003446* | *17615863* | *PADI3 | PADI4* | *N* | *N* | *N* | *Immunochip* |
| *rs745339* | *17616132* | *PADI3 | PADI4* | *Y* | *Y* | *Y* | *Immunochip* |
| *rs735280* | *17616184* | *PADI3 | PADI4* | *Y* | *Y* | *Y* | *Immunochip* |
| *rs11587669* | *17616259* | *PADI3 | PADI4* | *Y* | *Y* | *Y* | *Immunochip* |
| *rs74061529* | *17616369* | *PADI3 | PADI4* | *Y* | *Y* | *Y* | *Immunochip* |
| *rs11588418* | *17616445* | *PADI3 | PADI4* | *Y* | *Y* | *Y* | *Immunochip* |
| *rs72633804* | *17617024* | *PADI3 | PADI4* | *Y* | *Y* | *Y* | *Immunochip* |
| *rs115139898* | *17617268* | *PADI3 | PADI4* | *N* | *N* | *N* | *Immunochip* |
| *rs72633805* | *17617471* | *PADI3 | PADI4* | *Y* | *Y* | *Y* | *Immunochip* |
| *rs114612256* | *17617749* | *PADI3 | PADI4* | *N* | *N* | *N* | *Immunochip* |
| *rs55813674* | *17617887* | *PADI3 | PADI4* | *Y* | *Y* | *Y* | *Immunochip* |
| *rs72633806* | *17617943* | *PADI3 | PADI4* | *Y* | *Y* | *Y* | *Immunochip* |
| *rs115395508* | *17618341* | *PADI3 | PADI4* | *N* | *Y* | *Y* | *Immunochip* |
| *rs2501776* | *17619279* | *PADI3 | PADI4* | *Y* | *Y* | *Y* | *Immunochip* |
| *rs12410265* | *17620061* | *PADI3 | PADI4* | *Y* | *Y* | *Y* | *Immunochip* |
| *rs74061532* | *17620261* | *PADI3 | PADI4* | *Y* | *Y* | *Y* | *Immunochip* |
| *rs114437218* | *17620391* | *PADI3 | PADI4* | *N* | *Y* | *Y* | *Immunochip* |
| *rs2501777* | *17620851* | *PADI3 | PADI4* | *Y* | *Y* | *Y* | *Immunochip* |
| *rs11811933* | *17621327* | *PADI3 | PADI4* | *Y* | *Y* | *Y* | *Immunochip* |
| *rs7417622* | *17621758* | *PADI3 | PADI4* | *Y* | *Y* | *Y* | *Immunochip* |
| *rs7552802* | *17622148* | *PADI3 | PADI4* | *Y* | *Y* | *Y* | *Immunochip* |
| *rs7548900* | *17622654* | *PADI3 | PADI4* | *Y* | *Y* | *Y* | *Immunochip* |
| *rs7542629* | *17622897* | *PADI3 | PADI4* | *Y* | *Y* | *Y* | *Immunochip* |
| *rs72633812* | *17623140* | *PADI3 | PADI4* | *Y* | *Y* | *Y* | *Immunochip* |
| *rs12059651* | *17623430* | *PADI3 | PADI4* | *Y* | *Y* | *Y* | *Immunochip* |
| *rs115410341* | *17624203* | *PADI3 | PADI4* | *N* | *N* | *N* | *Immunochip* |
| *rs7411021* | *17624839* | *PADI3 | PADI4* | *Y* | *Y* | *Y* | *Immunochip* |
| *rs12048953* | *17624849* | *PADI3 | PADI4* | *Y* | *Y* | *Y* | *Immunochip* |
| *rs6657142* | *17624924* | *PADI3 | PADI4* | *Y* | *Y* | *Y* | *Immunochip* |
| *rs7517203* | *17625035* | *PADI3 | PADI4* | *N* | *N* | *Y* | *Immunochip* |
| *rs11801319* | *17625116* | *PADI3 | PADI4* | *Y* | *Y* | *Y* | *Immunochip* |
| *rs6586516* | *17625531* | *PADI3 | PADI4* | *Y* | *Y* | *Y* | *Immunochip* |
| *rs2501778* | *17627411* | *PADI3 | PADI4* | *Y* | *Y* | *Y* | *Immunochip* |
| *rs2501779* | *17627622* | *PADI3 | PADI4* | *Y* | *Y* | *Y* | *Immunochip* |
| *rs6688265* | *17627780* | *PADI3 | PADI4* | *Y* | *Y* | *Y* | *Immunochip* |
| *rs9435764* | *17627844* | *PADI3 | PADI4* | *Y* | *Y* | *Y* | *Immunochip* |
| *rs115451617* | *17628851* | *PADI3 | PADI4* | *N* | *Y* | *N* | *Immunochip* |
| *rs2501781* | *17628964* | *PADI3 | PADI4* | *Y* | *Y* | *Y* | *Immunochip* |
| *rs2477141* | *17629045* | *PADI3 | PADI4* | *Y* | *Y* | *Y* | *Immunochip* |
| *rs2501782* | *17629530* | *PADI3 | PADI4* | *Y* | *Y* | *Y* | *Immunochip* |
| *imm_1_17502331* | *17629744* | *PADI3 | PADI4* | *N* | *N* | *Y* | *Immunochip* |
| *rs2501783* | *17629980* | *PADI3 | PADI4* | *Y* | *Y* | *Y* | *Immunochip* |
| *rs6664258* | *17630115* | *PADI3 | PADI4* | *Y* | *Y* | *Y* | *Immunochip* |
| *rs2501784* | *17630193* | *PADI3 | PADI4* | *Y* | *Y* | *Y* | *Immunochip* |
| *rs2501785* | *17630211* | *PADI3 | PADI4* | *Y* | *Y* | *Y* | *Immunochip* |
| *rs111556847* | *17630269* | *PADI3 | PADI4* | *N* | *N* | *Y* | *Immunochip* |
| *rs2477138* | *17630305* | *PADI3 | PADI4* | *Y* | *Y* | *Y* | *Immunochip* |
| *rs2501786* | *17630372* | *PADI3 | PADI4* | *Y* | *Y* | *Y* | *Immunochip* |
| *rs2477137* | *17630605* | *PADI3 | PADI4* | *Y* | *Y* | *Y* | *Immunochip* |
| *rs2501787* | *17630727* | *PADI3 | PADI4* | *Y* | *Y* | *Y* | *Immunochip* |
| *rs72633823* | *17630779* | *PADI3 | PADI4* | *Y* | *Y* | *Y* | *Immunochip* |
| *rs12401287* | *17630838* | *PADI3 | PADI4* | *Y* | *Y* | *Y* | *Immunochip* |
| *rs4625336* | *17630964* | *PADI3 | PADI4* | *Y* | *Y* | *Y* | *Immunochip* |
| *rs2501789* | *17631022* | *PADI3 | PADI4* | *Y* | *Y* | *Y* | *Immunochip* |
| *rs12402270* | *17631023* | *PADI3 | PADI4* | *Y* | *Y* | *Y* | *Immunochip* |
| *rs11800611* | *17631118* | *PADI3 | PADI4* | *Y* | *Y* | *Y* | *Immunochip* |
| *rs877551* | *17631353* | *PADI3 | PADI4* | *Y* | *Y* | *Y* | *Immunochip* |
| *rs79907974* | *17631406* | *PADI3 | PADI4* | *Y* | *Y* | *Y* | *Immunochip* |
| *rs877553* | *17631445* | *PADI3 | PADI4* | *Y* | *Y* | *Y* | *Immunochip* |
| *rs11800688* | *17631652* | *PADI3 | PADI4* | *Y* | *Y* | *Y* | *Immunochip* |
| *rs34051324* | *17631810* | *PADI3 | PADI4* | *Y* | *Y* | *Y* | *Immunochip* |
| *rs78138254* | *17631911* | *PADI3 | PADI4* | *Y* | *Y* | *Y* | *Immunochip* |
| *rs112208664* | *17632010* | *PADI3 | PADI4* | *Y* | *Y* | *Y* | *Immunochip* |
| *rs2501793* | *17632237* | *PADI3 | PADI4* | *N* | *N* | *Y* | *Immunochip* |
| *rs72633828* | *17632251* | *PADI3 | PADI4* | *Y* | *Y* | *Y* | *Immunochip* |
| *rs74058715* | *17632345* | *PADI3 | PADI4* | *Y* | *Y* | *Y* | *Immunochip* |
| *rs1886303* | *17632382* | *PADI3 | PADI4* | *Y* | *Y* | *Y* | *Immunochip* |
| *rs34324150* | *17632464* | *PADI3 | PADI4* | *Y* | *Y* | *Y* | *Immunochip* |
| *rs2501794* | *17632600* | *PADI3 | PADI4* | *N* | *N* | *Y* | *Immunochip* |
| *rs11579504* | *17633246* | *PADI3 | PADI4* | *Y* | *Y* | *Y* | *Immunochip* |
| *rs2501795* | *17633499* | *PADI3 | PADI4* | *Y* | *Y* | *Y* | *Immunochip* |
| *rs2477134* | *17633572* | *PADI3 | PADI4* | *Y* | *Y* | *Y* | *Immunochip* |
| *rs2477133* | *17633892* | *PADI3 | PADI4* | *N* | *N* | *Y* | *Immunochip* |
| *rs2501797* | *17634057* | *PADI3 | PADI4* | *N* | *N* | *Y* | *Immunochip* |
| *rs35381732* | *17634740* | *PADI4* | *N* | *N* | *Y* | *Immunochip* |
| *rs2501798* | *17635036* | *PADI4* | *N* | *N* | *N* | *Immunochip* |
| *rs2501799* | *17635072* | *PADI4* | *N* | *N* | *Y* | *Immunochip* |
| *rs2501800* | *17635237* | *PADI4* | *N* | *Y* | *N* | *Immunochip* |
| *rs1886301* | *17635411* | *PADI4* | *Y* | *Y* | *Y* | *Immunochip* |
| *rs13376691* | *17636141* | *PADI4* | *N* | *N* | *N* | *Immunochip* |
| *rs12569317* | *17636288* | *PADI4* | *Y* | *Y* | *Y* | *Immunochip* |
| *rs2501801* | *17636363* | *PADI4* | *Y* | *Y* | *Y* | *Immunochip* |
| *rs2501802* | *17636719* | *PADI4* | *N* | *N* | *N* | *Immunochip* |
| *rs4920591* | *17636860* | *PADI4* | *Y* | *Y* | *Y* | *Immunochip* |
| *rs116063462* | *17639010* | *PADI4* | *N* | *N* | *Y* | *Immunochip* |
| *rs35943476* | *17640368* | *PADI4* | *Y* | *N* | *Y* | *Immunochip* |
| *rs115109055* | *17640731* | *PADI4* | *N* | *N* | *Y* | *Immunochip* |
| *rs2501806* | *17640784* | *PADI4* | *Y* | *Y* | *Y* | *Immunochip* |
| *rs2501807* | *17641259* | *PADI4* | *Y* | *Y* | *Y* | *Immunochip* |
| *rs2501808* | *17641436* | *PADI4* | *Y* | *Y* | *Y* | *Immunochip* |
| *rs1886300* | *17641684* | *PADI4* | *Y* | *Y* | *Y* | *Immunochip* |
| *rs11581515* | *17641932* | *PADI4* | *N* | *N* | *N* | *Immunochip* |
| *rs76286252* | *17642129* | *PADI4* | *Y* | *N* | *Y* | *Immunochip* |
| *rs9970772* | *17642273* | *PADI4* | *Y* | *Y* | *Y* | *Immunochip* |
| *rs6673392* | *17643290* | *PADI4* | *N* | *N* | *N* | *Immunochip* |
| *rs6673715* | *17643572* | *PADI4* | *Y* | *Y* | *Y* | *Immunochip* |
| *rs34298414* | *17643777* | *PADI4* | *Y* | *Y* | *Y* | *Immunochip* |
| *rs10788662* | *17644587* | *PADI4* | *N* | *N* | *N* | *Immunochip* |
| *rs77074876* | *17645082* | *PADI4* | *N* | *N* | *Y* | *Immunochip* |
| *rs71575826* | *17645185* | *PADI4* | *N* | *N* | *N* | *Immunochip* |
| *rs2147333* | *17646526* | *PADI4* | *Y* | *Y* | *Y* | *Immunochip* |
| *rs2147332* | *17646694* | *PADI4* | *Y* | *Y* | *Y* | *Immunochip* |
| *rs4920592* | *17647920* | *PADI4* | *Y* | *Y* | *Y* | *Immunochip* |
| *rs115457674* | *17647986* | *PADI4* | *N* | *N* | *Y* | *Immunochip* |
| *rs10888018* | *17649358* | *PADI4* | *Y* | *Y* | *Y* | *Immunochip* |
| *rs11203357* | *17649497* | *PADI4* | *Y* | *Y* | *Y* | *Immunochip* |
| *rs10888020* | *17649614* | *PADI4* | *Y* | *Y* | *Y* | *Immunochip* |
| *rs12217055* | *17650531* | *PADI4* | *N* | *N* | *N* | *Immunochip* |
| *rs11203359* | *17650619* | *PADI4* | *Y* | *Y* | *Y* | *Immunochip* |
| *rs4920593* | *17650650* | *PADI4* | *N* | *N* | *N* | *Immunochip* |
| *rs12030939* | *17650818* | *PADI4* | *Y* | *Y* | *Y* | *Immunochip* |
| *rs6692262* | *17651230* | *PADI4* | *Y* | *Y* | *Y* | *Immunochip* |
| *rs1204895* | *17651628* | *PADI4* | *Y* | *Y* | *Y* | *Immunochip* |
| *rs13375202* | *17651637* | *PADI4* | *Y* | *Y* | *Y* | *Immunochip* |
| *rs11203360* | *17651694* | *PADI4* | *Y* | *Y* | *Y* | *Immunochip* |
| *rs4920594* | *17651926* | *PADI4* | *Y* | *Y* | *Y* | *Immunochip* |
| *rs1924552* | *17653189* | *PADI4* | *Y* | *Y* | *Y* | *Immunochip* |
| *rs1204894* | *17653954* | *PADI4* | *Y* | *Y* | *Y* | *Immunochip* |
| *rs72633848* | *17654375* | *PADI4* | *Y* | *Y* | *Y* | *Immunochip* |
| *rs1748041* | *17655407* | *PADI4* | *Y* | *Y* | *Y* | *Immunochip* |
| *rs1635599* | *17655648* | *PADI4* | *Y* | *Y* | *Y* | *Immunochip* |
| *rs116727300* | *17655871* | *PADI4* | *N* | *N* | *N* | *Immunochip* |
| *rs11203365* | *17656496* | *PADI4* | *Y* | *Y* | *Y* | *Immunochip* |
| *rs12035959* | *17656635* | *PADI4* | *Y* | *Y* | *Y* | *Immunochip* |
| *rs12034419* | *17656766* | *PADI4* | *Y* | *Y* | *Y* | *Immunochip* |
| *rs11583124* | *17657004* | *PADI4* | *N* | *N* | *N* | *Immunochip* |
| *rs1748038* | *17657123* | *PADI4* | *Y* | *Y* | *Y* | *Immunochip* |
| *rs1635598* | *17657321* | *PADI4* | *Y* | *Y* | *Y* | *Immunochip* |
| *rs11203366* | *17657534* | *PADI4* | *Y* | *Y* | *Y* | *Immunochip* |
| *rs77182432* | *17657536* | *PADI4* | *N* | *N* | *Y* | *Immunochip* |
| *rs35809521* | *17657607* | *PADI4* | *N* | *N* | *Y* | *Immunochip* |
| *rs11203367* | *17657616* | *PADI4* | *Y* | *Y* | *Y* | *Immunochip* |
| *rs35809798* | *17657986* | *PADI4* | *Y* | *Y* | *Y* | *Immunochip* |
| *rs12035646* | *17658562* | *PADI4* | *Y* | *Y* | *Y* | *Immunochip* |
| *rs12033604* | *17658697* | *PADI4* | *Y* | *Y* | *Y* | *Immunochip* |
| *rs4506507* | *17659176* | *PADI4* | *Y* | *Y* | *Y* | *Immunochip* |
| *rs1748036* | *17659288* | *PADI4* | *Y* | *Y* | *Y* | *Immunochip* |
| *rs71644036* | *17659386* | *PADI4* | *Y* | *N* | *Y* | *Immunochip* |
| *rs1635597* | *17659556* | *PADI4* | *Y* | *Y* | *Y* | *Immunochip* |
| *rs115059002* | *17659563* | *PADI4* | *N* | *N* | *N* | *Immunochip* |
| *rs3795214* | *17659813* | *PADI4* | *Y* | *Y* | *Y* | *Immunochip* |
| *rs78540609* | *17660352* | *PADI4* | *N* | *N* | *Y* | *Immunochip* |
| *rs34309058* | *17660468* | *PADI4* | *N* | *N* | *N* | *Immunochip* |
| *rs874881* | *17660499* | *PADI4* | *Y* | *Y* | *Y* | *Immunochip* |
| *rs57744451* | *17660601* | *PADI4* | *Y* | *Y* | *Y* | *Immunochip* |
| *rs1548323* | *17660697* | *PADI4* | *Y* | *Y* | *Y* | *Immunochip* |
| *rs1555658* | *17661128* | *PADI4* | *Y* | *Y* | *Y* | *Immunochip* |
| *rs6586526* | *17661259* | *PADI4* | *N* | *N* | *Y* | *Immunochip* |
| *imm_1_17533955* | *17661368* | *PADI4* | *N* | *N* | *Y* | *Immunochip* |
| *rs1635594* | *17661805* | *PADI4* | *Y* | *Y* | *Y* | *Immunochip* |
| *rs11585797* | *17661934* | *PADI4* | *Y* | *N* | *Y* | *Immunochip* |
| *rs1748035* | *17661996* | *PADI4* | *Y* | *Y* | *Y* | *Immunochip* |
| *rs113475583* | *17662433* | *PADI4* | *Y* | *Y* | *Y* | *Immunochip* |
| *rs1748034* | *17662541* | *PADI4* | *Y* | *Y* | *Y* | *Immunochip* |
| *rs2240340* | *17662639* | *PADI4* | *Y* | *Y* | *Y* | *Immunochip/TaqMan* |
| *rs1621005* | *17662751* | *PADI4* | *Y* | *Y* | *Y* | *Immunochip* |
| *rs1748032* | *17662804* | *PADI4* | *Y* | *Y* | *Y* | *Immunochip* |
| *rs1748031* | *17662907* | *PADI4* | *Y* | *Y* | *Y* | *Immunochip* |
| *rs117561088* | *17663128* | *PADI4* | *N* | *N* | *N* | *Immunochip* |
| *rs78167936* | *17663203* | *PADI4* | *Y* | *N* | *Y* | *Immunochip* |
| *rs1748030* | *17663291* | *PADI4* | *Y* | *Y* | *Y* | *Immunochip* |
| *rs1748029* | *17663338* | *PADI4* | *Y* | *Y* | *Y* | *Immunochip* |
| *rs1635592* | *17663407* | *PADI4* | *Y* | *Y* | *Y* | *Immunochip* |
| *rs1635591* | *17663630* | *PADI4* | *Y* | *Y* | *Y* | *Immunochip* |
| *rs1748027* | *17663930* | *PADI4* | *Y* | *Y* | *Y* | *Immunochip* |
| *rs1635589* | *17664139* | *PADI4* | *Y* | *Y* | *Y* | *Immunochip* |
| *rs12089685* | *17664480* | *PADI4* | *N* | *N* | *N* | *Immunochip* |
| *rs11588132* | *17664615* | *PADI4* | *N* | *N* | *N* | *Immunochip* |
| *rs1635586* | *17664770* | *PADI4* | *Y* | *Y* | *Y* | *Immunochip* |
| *rs1635583* | *17665003* | *PADI4* | *Y* | *Y* | *Y* | *Immunochip* |
| *rs2477152* | *17665064* | *PADI4* | *Y* | *Y* | *Y* | *Immunochip* |
| *rs1635581* | *17665175* | *PADI4* | *Y* | *Y* | *Y* | *Immunochip* |
| *rs1635580* | *17665293* | *PADI4* | *Y* | *Y* | *Y* | *Immunochip* |
| *rs1635579* | *17665401* | *PADI4* | *Y* | *Y* | *Y* | *Immunochip* |
| *rs72637416* | *17665527* | *PADI4* | *N* | *N* | *N* | *Immunochip* |
| *rs35489932* | *17665664* | *PADI4* | *Y* | *N* | *Y* | *Immunochip* |
| *rs942460* | *17665685* | *PADI4* | *Y* | *Y* | *Y* | *Immunochip* |
| *rs12039455* | *17665781* | *PADI4* | *Y* | *Y* | *N* | *Immunochip* |
| *rs16825533* | *17665881* | *PADI4* | *Y* | *Y* | *Y* | *Immunochip* |
| *rs35442836* | *17666004* | *PADI4* | *Y* | *N* | *Y* | *Immunochip* |
| *rs1635578* | *17666095* | *PADI4* | *Y* | *Y* | *Y* | *Immunochip* |
| *rs1635577* | *17666139* | *PADI4* | *Y* | *Y* | *Y* | *Immunochip* |
| *imm_1_17538794* | *17666207* | *PADI4* | *N* | *N* | *Y* | *Immunochip* |
| *rs11203368* | *17666508* | *PADI4* | *Y* | *Y* | *Y* | *Immunochip* |
| *rs1748023* | *17666584* | *PADI4* | *Y* | *Y* | *Y* | *Immunochip* |
| *rs1748022* | *17666714* | *PADI4* | *Y* | *Y* | *Y* | *Immunochip* |
| *rs34965564* | *17666747* | *PADI4* | *Y* | *N* | *Y* | *Immunochip* |
| *rs1635574* | *17667106* | *PADI4* | *N* | *N* | *N* | *Immunochip* |
| *rs1748021* | *17668270* | *PADI4* | *Y* | *Y* | *Y* | *Immunochip* |
| *rs16825565* | *17668508* | *PADI4* | *N* | *N* | *Y* | *Immunochip* |
| *rs35903413* | *17668563* | *PADI4* | *Y* | *N* | *Y* | *Immunochip* |
| *rs1748020* | *17668609* | *PADI4* | *Y* | *Y* | *Y* | *Immunochip* |
| *rs1748019* | *17668976* | *PADI4* | *Y* | *Y* | *Y* | *Immunochip* |
| *rs1748018* | *17669202* | *PADI4* | *Y* | *Y* | *Y* | *Immunochip* |
| *rs6662651* | *17669911* | *PADI4* | *N* | *N* | *N* | *Immunochip* |
| *rs41266003* | *17672642* | *PADI4* | *Y* | *N* | *Y* | *Immunochip* |
| *rs1635571* | *17672729* | *PADI4* | *Y* | *Y* | *Y* | *Immunochip* |
| *rs2301888* | *17672730* | *PADI4* | *Y* | *Y* | *Y* | *Immunochip* |
| *rs72637418* | *17672989* | *PADI4* | *Y* | *N* | *Y* | *Immunochip* |
| *rs35210719* | *17673102* | *PADI4* | *Y* | *Y* | *Y* | *Immunochip* |
| *rs6683037* | *17673259* | *PADI4* | *Y* | *Y* | *Y* | *Immunochip* |
| *rs2240339* | *17674108* | *PADI4* | *Y* | *Y* | *Y* | *Immunochip* |
| *rs2240338* | *17674185* | *PADI4* | *Y* | *Y* | *Y* | *Immunochip* |
| *rs2240337* | *17674222* | *PADI4* | *Y* | *Y* | *Y* | *Immunochip* |
| *rs35287029* | *17674358* | *PADI4* | *Y* | *N* | *Y* | *Immunochip* |
| *rs2240336* | *17674402* | *PADI4* | *Y* | *Y* | *Y* | *Immunochip* |
| *rs2240335* | *17674537* | *PADI4* | *Y* | *Y* | *Y* | *Immunochip* |
| *rs1635570* | *17674601* | *PADI4* | *Y* | *Y* | *Y* | *Immunochip* |
| *rs12746252* | *17674717* | *PADI4* | *Y* | *N* | *Y* | *Immunochip* |
| *rs12746451* | *17674821* | *PADI4* | *Y* | *Y* | *Y* | *Immunochip* |
| *rs1408424* | *17674868* | *PADI4* | *Y* | *Y* | *Y* | *Immunochip* |
| *rs12731545* | *17675012* | *PADI4* | *Y* | *N* | *Y* | *Immunochip* |
| *rs12754590* | *17675121* | *PADI4* | *Y* | *N* | *Y* | *Immunochip* |
| *rs3795213* | *17675197* | *PADI4* | *Y* | *Y* | *Y* | *Immunochip* |
| *rs35095098* | *17675393* | *PADI4* | *Y* | *N* | *Y* | *Immunochip* |
| *rs71644039* | *17675713* | *PADI4* | *Y* | *N* | *Y* | *Immunochip* |
| *rs1613767* | *17675889* | *PADI4* | *Y* | *Y* | *Y* | *Immunochip* |
| *rs112930800* | *17676236* | *PADI4* | *N* | *N* | *N* | *Immunochip* |
| *rs1748014* | *17676343* | *PADI4* | *N* | *N* | *N* | *Immunochip* |
| *rs4450060* | *17676630* | *PADI4* | *N* | *N* | *N* | *Immunochip* |
| *rs12752435* | *17676740* | *PADI4* | *N* | *N* | *N* | *Immunochip* |
| *rs72637425* | *17676774* | *PADI4* | *N* | *N* | *N* | *Immunochip* |
| *rs72637426* | *17676834* | *PADI4* | *N* | *N* | *N* | *Immunochip* |
| *rs12752914* | *17676969* | *PADI4* | *N* | *N* | *N* | *Immunochip* |
| *rs12725408* | *17677040* | *PADI4* | *N* | *N* | *Y* | *Immunochip* |
| *rs11203369* | *17677069* | *PADI4* | *N* | *Y* | *N* | *Immunochip* |
| *rs35023063* | *17677196* | *PADI4* | *N* | *N* | *N* | *Immunochip* |
| *rs71512933* | *17677270* | *PADI4* | *N* | *N* | *Y* | *Immunochip* |
| *rs71512934* | *17677362* | *PADI4* | *N* | *N* | *Y* | *Immunochip* |
| *rs12083961* | *17677755* | *PADI4* | *N* | *N* | *Y* | *Immunochip* |
| *rs67858127* | *17678599* | *PADI4* | *Y* | *Y* | *Y* | *Immunochip* |
| *rs114455989* | *17678662* | *PADI4* | *N* | *N* | *N* | *Immunochip* |
| *rs12042956* | *17678713* | *PADI4* | *Y* | *Y* | *Y* | *Immunochip* |
| *rs79331366* | *17678790* | *PADI4* | *Y* | *N* | *Y* | *Immunochip* |
| *rs4920600* | *17681424* | *PADI4* | *Y* | *Y* | *Y* | *TaqMan assay* |
| *rs1408422* | *17690915* | *PADI4 | PADI6* | *N* | *N* | *N* | *Immunochip* |
| *rs7538597* | *17691983* | *PADI4 | PADI6* | *N* | *N* | *Y* | *Immunochip* |
| *rs2526839* | *17717312* | *PADI6* | *Y* | *Y* | *Y* | *Immunochip* |
| *rs7538876* | *17722363* | *PADI6* | *Y* | *Y* | *Y* | *Immunochip* |

**Table S2.** Haplotype frequencies and meta-analysis of PADI4 polymorphisms in the MyEIRA study by ACPA status

| Block | ACPA status | Ethnicity | Haplotype Freq. | Case, Control Ratio Counts | Case, Control Frequencies | X^2^ | P Value | OR (95% CI) | Meta-analysis | P_het_ | I^2^(%) |
| --- | --- | --- | --- | --- | --- | --- | --- | --- | --- | --- | --- |
| ACGG | ACPA+ | Malay | 0.502 | 300.6 : 323.4, 984.3 : 951.7 | 0.482, 0.508 | 1.336 | 0.2478 | 0.90 (0.75-1.08) | 0.92 (0.81-1.04)  Z=1.36 (P=0.17) | 0.71 | 0 |
|  |  | Chinese | 0.568 | 191.2 : 140.8, 224.4 : 175.6 | 0.576, 0.561 | 0.162 | 0.6875 | 1.06 (0.79-1.43) |
|  |  | Indian | 0.423 | 201.2 : 298.8, 246.8 : 313.2 | 0.402, 0.441 | 1.586 | 0.2079 | 0.85 (0.67-1.09) |
|  |  | Others | 0,464 | 56.1 : 67.9, 88.6 : 99.4 | 0.452, 0.471 | 0,105 | 0,7458 | 0.92 (0.58-1.44) |
|  |  |  |  |  |  |  |  |  |  |  |  |
|  | ACPA- | Malay | 0.507 | 210.2 : 213.8, 984.4 : 947.6 | 0.496, 0.510 | 0.261 | 0.6092 | 0.95 (0.77-1.17) | 0.90 (0.78-1.05)  Z=1.34 (p=0.18) | 0.70 | 0 |
|  |  | Chinese | 0.566 | 97.9 : 76.1, 226.7 : 173.3 | 0.563, 0.567 | 0.0090 | 0.9262 | 0.98 (0.69-1.41) |
|  |  | Indian | 0.418 | 92.8 : 155.2, 245.2 : 314.8 | 0.374, 0.438 | 2.877 | 0.0898 | 0.77 (0.57-1.05) |
|  |  | Others | 0,464 | 22.6 : 29.4, 88.6 : 99.4 | 0.435, 0.471 | 0,216 | 0,6419 | 0.88 (0.48-1.64) |
|  |  |  |  |  |  |  |  |  |  |  |  |
| ATAA | ACPA+ | Malay | 0.059 | 29.3 : 594.7, 121.0 : 1815.0 | 0.047, 0.062 | 2.042 | 0.153 | 0.73 (0.48-1.11) | 0.77 (0.57-1.05)  Z=1.65 (P=0.10) | 0.57 | 0 |
|  |  | Chinese | 0.066 | 19.0 : 313.0, 29.0 : 371.0 | 0.057, 0.072 | 0.69 | 0.406 | 0.78 (0.43-1.41) |
|  |  | Indian | 0.014 | 5.1 : 494.9, 10.0 : 550.0 | 0.010, 0.018 | 1.123 | 0.2893 | 0.56 (0.19-1.64) |
|  |  | Others | 0,046 | 7.4 : 116.6, 7.0 : 181.0 | 0.059, 0.037 | 0,838 | 0,3599 | 1.55 (0.53-4.52) |
|  |  |  |  |  |  |  |  |  |  |  |  |
|  | ACPA- | Malay | 0.057 | 13.3 : 410.7, 121.2 : 1810.8 | 0.031, 0.063 | 6.323 | 0.0119 | **0.47 (0.26-0.85)** | **0.59 (0.39-0.90)**  **Z=2.47 (p=0.01**) | 0.43 | 0 |
|  |  | Chinese | 0.066 | 10.0 : 164.0, 28.0 : 372.0 | 0.057, 0.070 | 0.308 | 0.579 | 0.81 (0.38-1.71) |
|  |  | Indian | 0.016 | 4.3 : 243.7, 8.3 : 551.7 | 0.017, 0.015 | 0.064 | 0.801 | 1.13 (0.34-3.79) |
|  |  | Others | 0,029 | 0.0 : 52.0, 7.0 : 181.0 | 0.000, 0.037 | 1,994 | 0,1579 | 0.23 (0.01-4.10) |
|  |  |  |  |  |  |  |  |  |  |  |  |
| ATAG | ACPA+ | Malay | 0.280 | 195.1 : 428.9, 522.9 : 1413.1 | 0.313, 0.270 | 4.24 | 0.0395 | **1.23 (1.01-1.50)** | **1.91 (1.04-1.36)**  **Z=2.52 (p=0.01)** | 0.71 | 0 |
|  |  | Chinese | 0.270 | 90.5 : 241.5, 107.3 : 292.7 | 0.273, 0.268 | 0.019 | 0.8912 | 1.03 (0.75-1.43) |
|  |  | Indian | 0.398 | 213.7 : 286.3, 207.6 : 352.4 | 0.427, 0.371 | 3.552 | 0.0595 | 1.27 (0.99-1.62) |
|  |  | Others | 0,328 | 41.1 : 82.9, 61.1 : 126.9 | 0.332, 0.325 | 0,015 | 0,9011 | 1.03 (0.63-1.67) |
|  |  |  |  |  |  |  |  |  |  |  |  |
|  | ACPA- | Malay | 0.281 | 138.6 : 285.4, 522.5 : 1409.5 | 0.327, 0.270 | 5.482 | 0.0192 | **1.31 (1.05-1.65)** | **1.24 (1.05-1.45)**  **Z=2.60 (p=0.009)** | 0.81 | 0 |
|  |  | Chinese | 0.272 | 50.0 : 124.0, 106.0 : 294.0 | 0.287, 0.265 | 0.305 | 0.5808 | 1.12 (0.75-1.66) |
|  |  | Indian | 0.378 | 99.2 : 148.8, 205.8 : 354.2 | 0.400, 0.368 | 0.773 | 0.3794 | 1.14 (0.84-1.55) |
|  |  | Others | 0,345 | 21.4 : 30.6, 61.4 : 126.6 | 0.411, 0.327 | 1,277 | 0,2585 | 1.41 (0.75-2.65) |
|  |  |  |  |  |  |  |  |  |  |  |  |
| ATGG | ACPA+ | Malay | 0.096 | 68.0 : 556.0, 178.7 : 1757.3 | 0.109, 0.092 | 1.497 | 0.2212 | 1.20 (0.89-1.61) | 1.06 (0.83-1.34)  Z=0.45 (p=0.66) | 0.23 | 30 |
|  |  | Chinese | 0.046 | 15.1 : 316.9, 18.3 : 381.7 | 0.045, 0.046 | 0.0 | 0.9909 | 1.00 (0.50-2.02) |
|  |  | Indian | 0.044 | 22.5 : 477.5, 24.5 : 535.5 | 0.045, 0.044 | 0.0080 | 0.9291 | 1.03 (0.58-1.84) |
|  |  | Others | 0,094 | 7.0 : 117.0, 22.3 : 165.7 | 0.057, 0.118 | 3,347 | 0,0673 | 0.45 (0.19-1.09) |
|  |  |  |  |  |  |  |  |  |  |  |  |
|  | ACPA- | Malay | 0.092 | 40.6 : 383.4, 177.3 : 1754.7 | 0.096, 0.092 | 0.063 | 0.8011 | 1.06 (0.74-1.52) | 1.06 (0.80-1.40)  Z=0.40 (p=0.69) | 0.73 | 0 |
|  |  | Chinese | 0.046 | 8.1 : 165.9, 18.3 : 381.7 | 0.047, 0.046 | 0.0020 | 0.9634 | 1.02 (0.44-2.40) |
|  |  | Indian | 0.055 | 16.0 : 232.0, 28.3 : 531.7 | 0.065, 0.051 | 0.64 | 0.4238 | 1.31 (0.70-2.47) |
|  |  | Others | 0,108 | 4.0 : 48.0, 21.9 : 166.1 | 0.077, 0.117 | 0,665 | 0,4149 | 0.63 (0.21-1.91) |
|  |  |  |  |  |  |  |  |  |  |  |  |
| GTAG | ACPA+ | Malay | 0.041 | 22.4 : 601.6, 83.5 : 1852.5 | 0.036, 0.043 | 0.621 | 0.4306 | 0.81 (0.50-1.30) | 0.87 (0.64-1.17)  Z=0.92 (p=0.36) | 0.57 | 0 |
|  |  | Chinese | 0.041 | 12.4 : 319.6, 17.7 : 382.3 | 0.037, 0.044 | 0.209 | 0.6473 | 0.80 (0.38-1.68) |
|  |  | Indian | 0.060 | 27.2 : 472.8, 36.3 : 523.7 | 0.054, 0.065 | 0.524 | 0.4693 | 0.83 (0.50-1.39) |
|  |  | Others | 0,046 | 7.8 : 116.2, 6.6 : 181.4 | 0.063, 0.035 | 1,343 | 0,2466 | 1.78 (0.63-5.05) |
|  |  |  |  |  |  |  |  |  |  |  |  |
|  | ACPA- | Malay | 0.042 | 16.1 : 407.9, 83.0 : 1849.0 | 0.038, 0.043 | 0.223 | 0.6369 | 0.87 (0.51-1.51) | 1.03 (0.73-1.45)  Z=0.17 (p=0.87) | 0.74 | 0 |
|  |  | Chinese | 0.047 | 8.0 : 166.0, 19.0 : 381.0 | 0.046, 0.047 | 0.0060 | 0.9369 | 0.97 (0.41-2.25) |
|  |  | Indian | 0.074 | 20.2 : 227.8, 39.2 : 520.8 | 0.082, 0.070 | 0.338 | 0.5611 | 1.17 (0.67-2.05) |
|  |  | Others | 0,037 | 2.6 : 49.4, 6.3 : 181.7 | 0.051, 0.034 | 0,328 | 0,5669 | 1.86 (0.45-7.69) |

PADI4: peptidylarginine deiminase Type 4; MyEIRA: Malaysian Epidemiological Investigation of Rheumatoid Arthritis; X^2^: chi-square; OR (95% CI): odds ratio (95% confidence interval); P_het_ (p value for heterogeneity), I^2^=degree of heterogeneity expressed as percentage; RA: rheumatoid arthritis; ACPA+: anti-citrullinated protein antibody-positive; ACPA-: anti-citrullinated protein antibody-negative.

**Figure S1.** **Regional association plots with recombination rate on the PADI genes for the three major ethnic groups from MyEIRA study.** The graphs are centered on the most significant SNP in each ethnic group. The r^2^ values (the linkage disequilibrium between the most significant SNP and the rest of SNPs in the region) are calculated in MyEIRA materials, and the recombination rates are based on the CHB+JPT HapMap data. **(a)** Malay ethnic group, **(b)** Chinese ethnic group, and **(c)** Indian ethnic group.

1. Malay ethnic group


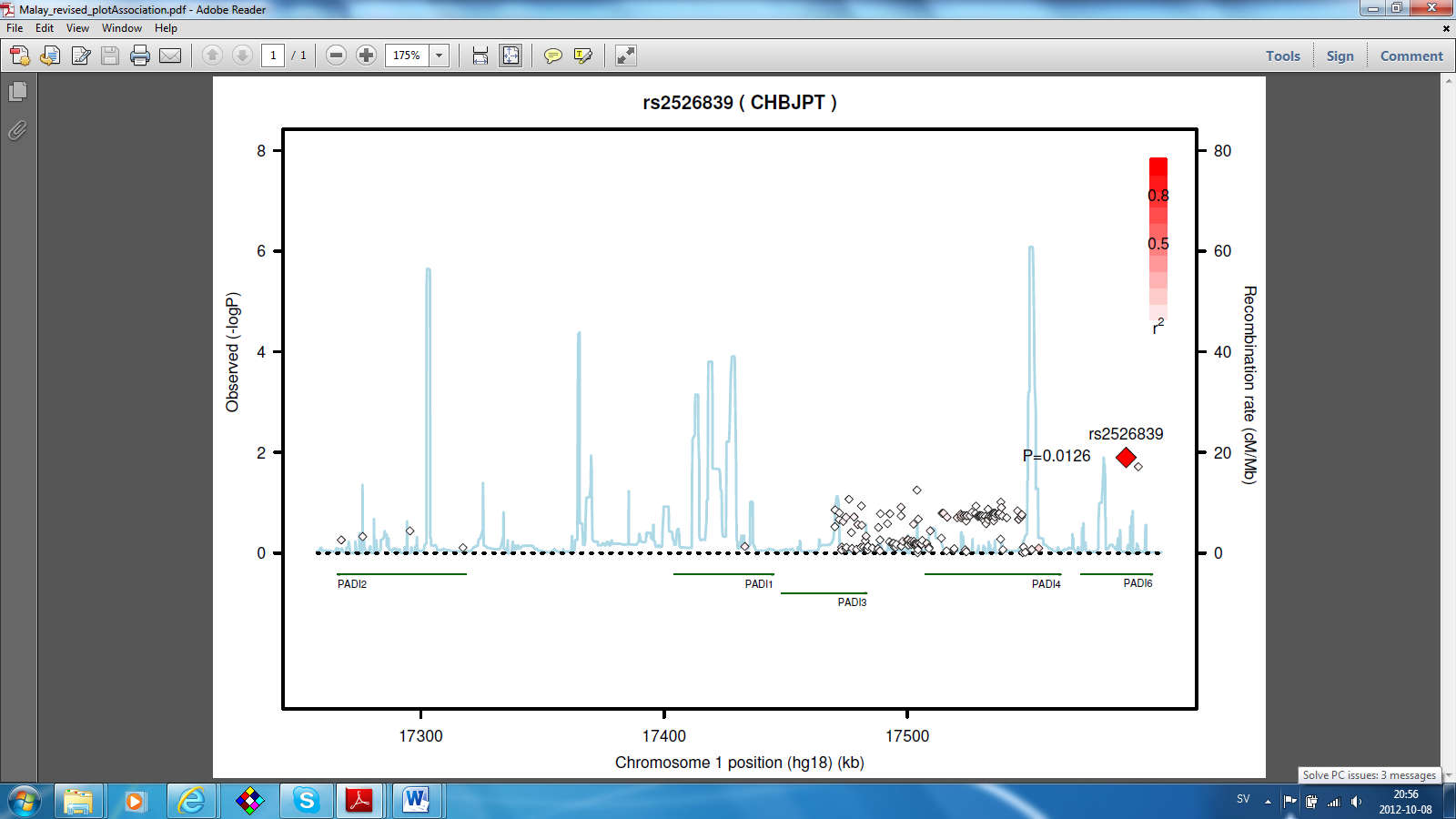


1. Chinese ethnic group


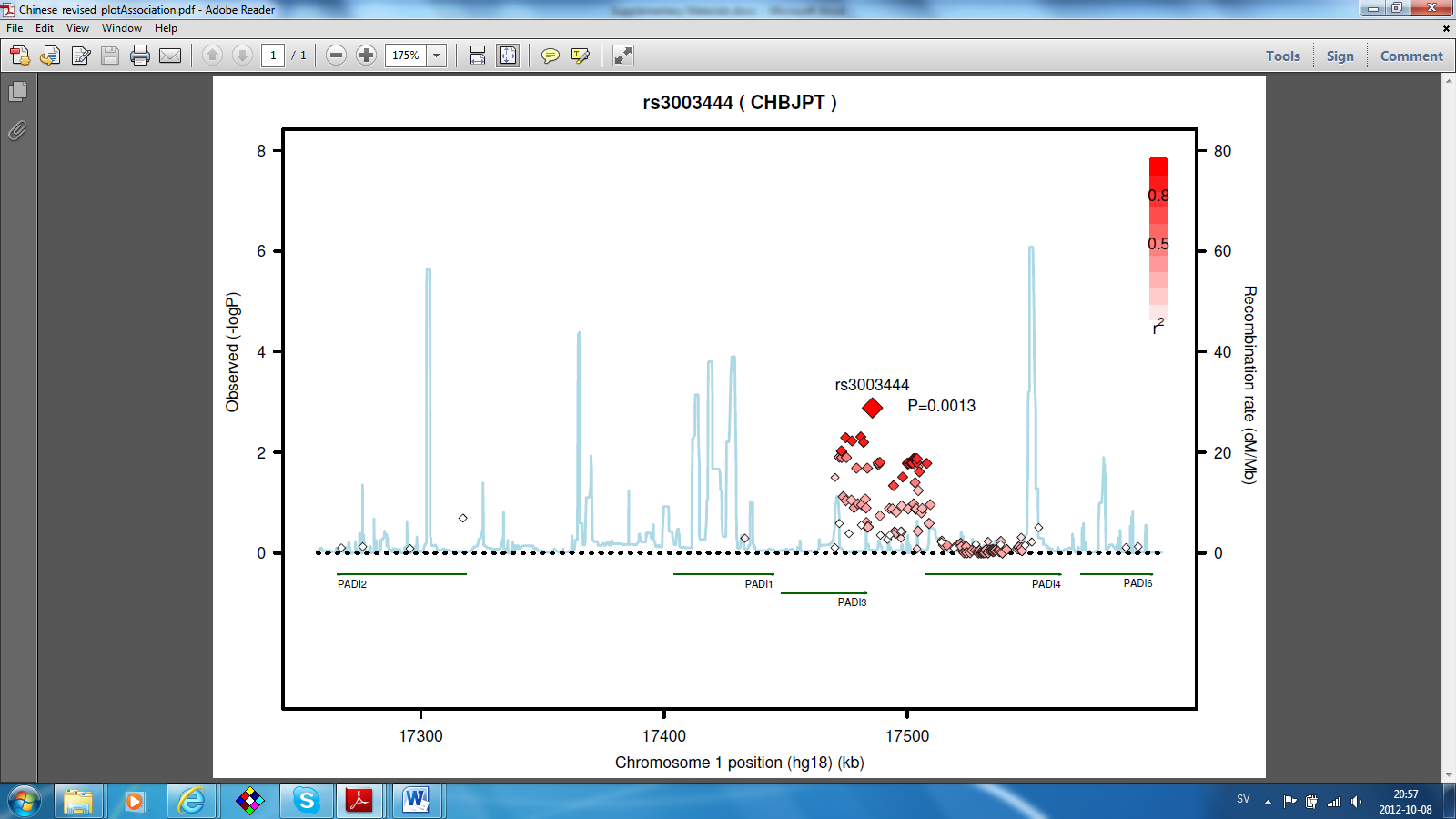


1. Indian ethnic group


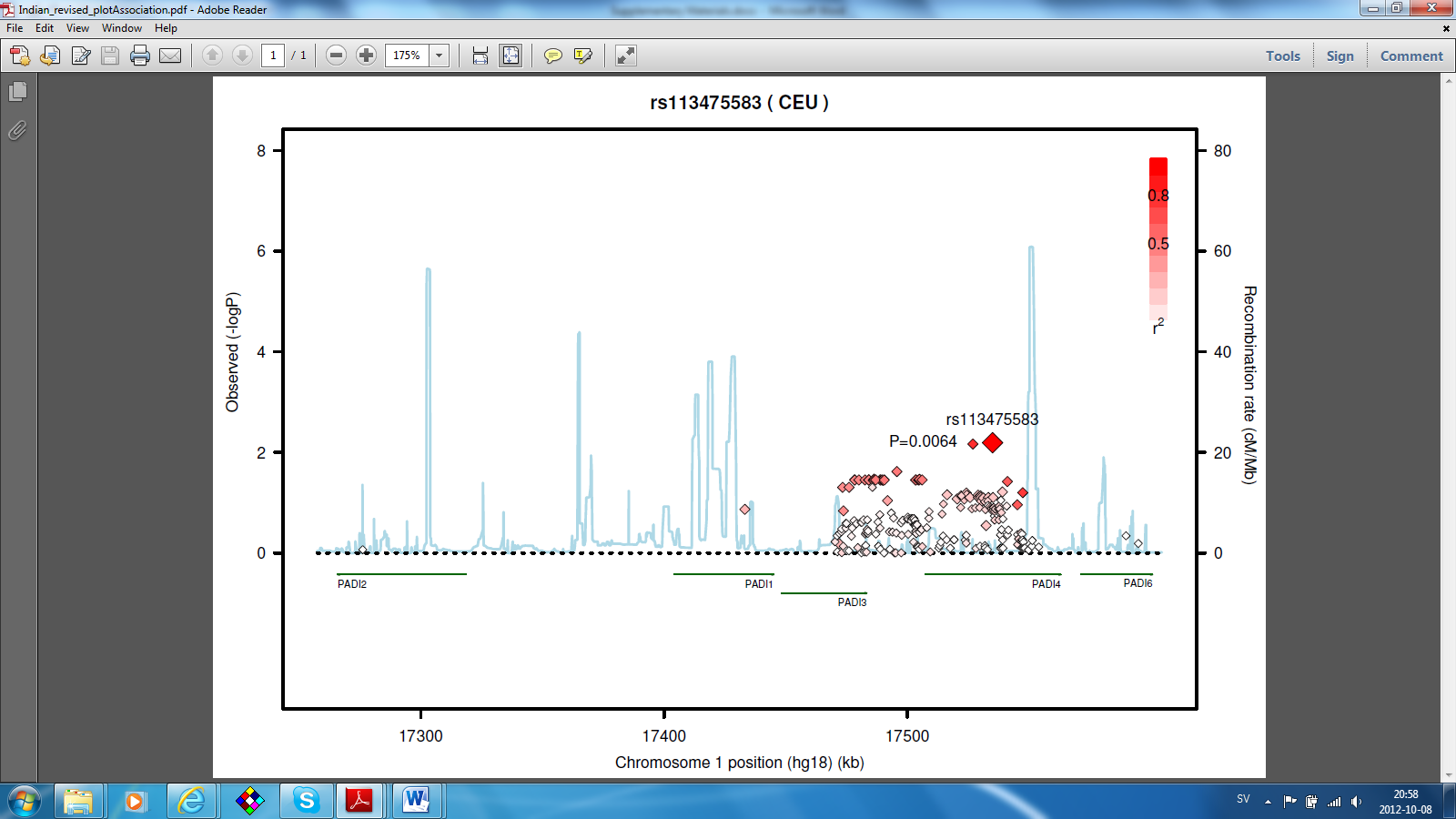

Supplement: Additional file 1 — Table S1 presenting a list of PADI SNPs investigated in the MyEIRA study population. A complete set of 320 SNPs selected from the PADI locus on Immunochip and from other studies. The SNPs were genotyped either using the TaqMan SNP genotyping assay (Applied Biosystems, USA) or by Illumina iSELECT HD custom genotyping array (Immunochip). Table S2 presenting the haplotype frequencies and meta-analysis of PADI4 polymorphisms in the MyEIRA study by ACPA status. The haplotype analysis and meta-analysis of PADI4 polymorphisms in the MyEIRA study were performed in different subsets of RA defined by ACPA status. Bold results indicate significant association between the PADI haplotypes and subsets of RA. Figure S1 showing the regional association plots with recombination rate on the PADI genes for the three major ethnic groups from the MyEIRA study. Regional association plots on the PADI genes including PADI1, PADI2, PADI3, PADI4 and PADI6 for the three major ethnic groups from MyEIRA study showing the peak association in each ethnic group. Graphs centered on the most significant SNP in each ethnic group. The r2 values (linkage disequilibrium between the most significant SNP and the rest of SNPs in the region) are calculated on the MyEIRA data and the recombination rates are based on the International HapMap CHB+JPT data. [file ar4093-S1.DOC]
